# SOX9 represses the human galectin-3 promoter in SW1353 cells: potential implications for osteoarthritis

**DOI:** 10.1038/s41598-026-50507-0

**Published:** 2026-05-05

**Authors:** Blanca Alba, Shivani Buddiga, Herbert Kaltner, Stefan Toegel, Sebastian Schmidt

**Affiliations:** 1 Department of Veterinary Sciences, Chair of Biochemistry and Chemistry, Ludwig-Maximilians-Universität Munich, Lena-Christ-Str. 48, 82152 Planegg-Martinsried, Germany; 2 Department of Orthopedics and Trauma Surgery, Karl Chiari Lab for Orthopaedic Biology, Medical University of Vienna, Waehringer Guertel 18-20, Vienna, 1090 Austria; 3 Ludwig Boltzmann Institute for Arthritis and Rehabilitation, Spitalgasse 23, Vienna, 1090 Austria

**Keywords:** Galectin-3, SOX9, Promoter analysis, Gene regulation, Osteoarthritis, Cell biology, Computational biology and bioinformatics, Genetics, Immunology, Molecular biology

## Abstract

Galectin-3 (Gal-3), a member of the β-galactoside-binding protein family, is critically involved in inflammation, extracellular matrix remodelling, and cartilage degeneration in osteoarthritis (OA). This study aims to elucidate the regulation of the human galectin-3 gene (*LGALS3*) promoter in SW1353 cells and its control by SOX transcription factors, known to be dysregulated during OA pathogenesis. We sought to identify key sequence elements in the *LGALS3* promoter responsible for its transcriptional activity and relevant transcription factors (TFs). Using luciferase reporter assays, we examined deletion variants of the 5’ region (-2638 bp to + 52 bp) and assessed their activation potential. We also identified potential transcription factor binding sites (TFBS) through *in silico* analyses and confirmed SOX9 binding in the − 93/+49 region by chromatin immunoprecipitation using HaloCHIP™. Functional assays revealed that the proximal promoter region (-97 bp to + 52 bp) is critical for reporter gene expression in SW1353 cells. This study demonstrates that the presence of *SOX9* leads to a dose-dependent decrease in *LGALS3* promoter activity in SW1353 cells. Consistent with this trend, SOX9 overexpression caused a modest reduction in endogenous *LGALS3* mRNA levels. Together, these findings highlight the importance of SOX TF interactions in regulating *LGALS3* expression and their potential role in chondrocytes.

**Supplementary Information:**

The online version contains supplementary material available at 10.1038/s41598-026-50507-0.

## Introduction

Galectins are an evolutionarily conserved family of glycan-binding proteins characterized by a conserved β-galactoside-binding carbohydrate recognition domain (CRD) consisting of a seven-amino acid sequence signature^1^. The 3D conformation of CRDs is “a β-sandwich described as a jelly-roll motif”^1^. Galectins are abundant across diverse species, present in vertebrates, protochordates, invertebrates, mushrooms, and viruses^2,3^.

Galectins can be classified according to the arrangement of their CRDs into three subgroups: prototype, tandem-repeat type, and chimera type. The chimera type galectin, represented solely by galectin-3 (gene name: *LGALS3*), possesses a unique structure combining a C-terminal CRD with an N-terminal domain rich in glycine-proline repeats, which facilitates oligomerization^1^. Galectin-3 levels in patients’ sera are characteristic of both osteoarthritis (OA) and early rheumatoid arthritis, underscoring the relevance of understanding the regulatory mechanisms of *LGALS3* in both conditions^4–6^. In OA, a degenerative joint disease characterised by chronic, low-grade inflammation^7–9^, galectin-3 presence in articular chondrocytes correlated positively with the severity of cartilage destruction^10^. Furthermore, extracellular galectin-3 bound to the surface of OA chondrocytes and induced the expression of pro-inflammatory cytokines and matrix metalloproteases by activating the NF-kB pathway^10^. This cascade eventually resulted in the degradation of extracellular matrix in a 3D organoid model of OA chondrocytes^11^. However, the exact mechanisms underlying the observed upregulation of galectin-3 in human OA chondrocytes remain unexplored, underscoring the need for studies that deepen our understanding of its molecular regulation in this disease condition.

*LGALS3* expression can be regulated by transcriptional and epigenetic mechanisms^6,12,13^. Among these regulatory layers, transcriptional control is particularly relevant for understanding how *LGALS3* expression is altered during disease progression. In OA, extensive transcriptomic remodelling occurs in articular chondrocytes, including significant alterations in transcription factor expression^14^. Public RNA-seq datasets from human osteoarthritic cartilage, such as GSE114007, confirm that *LGALS3* is expressed in articular cartilage and reveal disease-associated changes in multiple transcription factor families.

Among the transcription factors affected in OA cartilage, members of the SOX (SRY-related HMG-box) family are of particular interest. There are 20 SOX transcription factors conserved in mammals. The consensus sequence recognized by most SOX transcription factors is 5’- (A/T)(A/T)CAA(A/T)G −3’^15^. SOX proteins can bend the DNA upon binding to allow for chromatin modelling. They are crucial in cell fate determination, differentiation, and development^15^. SOX9, widely recognised as ‘the master regulator of cartilage development’, is critical in cartilage homeostasis, activating the expression of extracellular matrix (ECM) components such as collagen type II (COL2A1) and aggrecan (ACAN)^16^. SOX9, together with SOX5 and SOX6, builds the SOX trio, which cooperatively governs chondrocyte-specific gene expression^16^. These three SOX proteins are shown to be downregulated in OA chondrocytes^17^, whereas another member, SOX4, is upregulated^18^, indicating a disease-associated imbalance within the SOX transcriptional network. SOX2, in contrast, is not detectably expressed in articular cartilage and has no known relevance in osteoarthritis pathology; it was included in this study solely as a mechanistic comparison within the SOX family. In addition, functional interactions between galectins and SOX-dependent signalling pathways have been described, as galectin-1 is reported to activate the β-catenin-SOX9 signalling cascade, thereby promoting tumour progression in colorectal cancer^19^.

Given the central role of SOX transcription factors in maintaining chondrocyte identity, their documented dysregulation in OA, and the extensive transcriptional remodelling observed in osteoarthritic cartilage, it is conceivable that altered SOX activity contributes to the aberrant expression of OA-associated genes, including *LGALS3.* We therefore hypothesized that members of the SOX family directly regulate *LGALS3* transcription in human chondrocytes.

Therefore, this study aims to identify the key regulatory elements of the *LGALS3* promoter and SOX-binding motifs and assess the impact of members of the SOX family on gene expression in SW1353 cells, a human chondrosarcoma-derived cell line commonly used as a chondrocyte model^20–22^. By analysing *LGALS3* promoter deletions, we identified the minimal promoter region with maximal activity. Overexpression experiments revealed that *SOX2* and *SOX9* dose-dependently reduced promoter activity to 10%, whereas *SOX4*, *SOX5*, and *SOX6* had no significant influence. This repression with *SOX2* and *SOX9* persisted even with the shortest promoter construct (−97/+52). This study is the first to demonstrate that SOX transcription factors directly regulate the *LGALS3* promoter, establishing a novel link between the SOX family and galectin-3 expression in chondrocytes.

## Results

### Deletion analysis of the *LGALS3* promoter region

The first aim of this work was to identify critical sequence regions responsible for mediating *LGALS3* gene expression in the SW1353 chondrocyte model. The sequence region we investigated spans between − 2638 and + 52 relative to the transcription start site (TSS). A series of promoter fragments were inserted into the promoter-less luciferase reporter vector pGL4.20, upstream of the luciferase gene, to generate the different pGL4.20-hGal3p reporter plasmids. The empty promoter-less vector (pGL4.20) served as a reference for calculating the promoter activities.

**Fig. 1:**
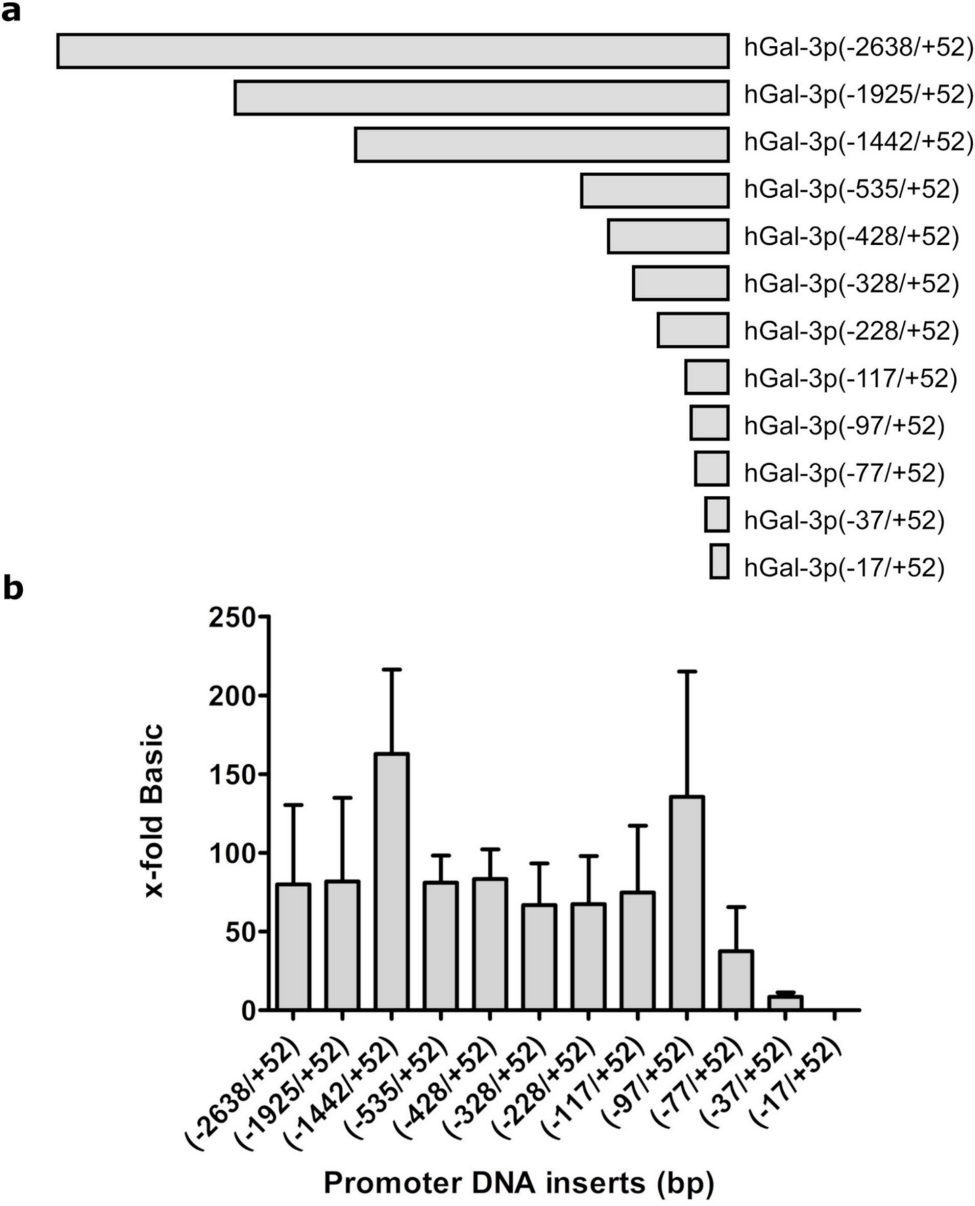
(**a**) The *LGALS3* full-length promoter (−2638/+52) and the deletions (−1925/+52), (−1442/+52), (−535/+52), (−428/+52), (−328/+52), (−228/+52), (−117/+52), (−97/+52), (−77/+52), (−37/+52), (−17/+52) are shown in a diagram drawn to scale. Positions are numbered according to the distance to the transcription start site (TSS), where negative means upstream and positive means downstream of the TSS. (**b**) Relative activity of *LGALS3* promoter sequence deletions in SW1353 cells. The activity of these deletions is shown as an x-fold increase relative to cells transfected with the promoter-less vector pGL4.20 (Basic). The graph shows the results’ mean ± SD of at least four biological replicates, each including three technical replicates, with error bars representing the standard deviation. *p < 0.05.

Figure 1a shows the relative lengths of each promoter deletion construct drawn to scale. Figure 1b shows maximum activities for the sequence stretches (−1442/+52) with 162.9-fold and (−97/+52) with 135.6-fold activity, and an activity decrease between (−1442/+52) and (−535/+52) to 81.1-fold (Fig. 1b). The promoter activity remains roughly constant between the consecutive deletion constructs from (−535/+52) to (−117/+52). When comparing the full-length promoter (−2638/+52) with the (−77/+52) deletion variant, the reporter assay activity decreased from 62.5-fold to 37.5-fold. Notably, there is an 81-fold increase in the promoter activity if we compare the relative activity of the full-length (−2638/+52) and (−1925/+52) variants to that of (−1442/+52), suggesting the presence of inhibitory regulatory elements within the distal − 2638/−1442 region, whose removal results in increased basal promoter activity. For the − 1442/−536 sequence and the observed loss in activation potential, regulatory sequences contributing to promoter activation may be deleted within this interval. The sustained high activity of the (−97/+52) sequence, followed by a stepwise decrease to 37.5-fold (−77/+52), 8.54-fold (−37/+52), and 0.06-fold (−17/+52) indicates that essential positive regulatory elements are located within the proximal − 97/+52 region. The progressive reduction in activity upon successive deletions suggests that this region contains critical cis-regulatory sequences required for maintaining basal promoter activity. *In silico* motif analysis of the proximal − 97/+52 region revealed a GC-rich architecture with several predicted transcription factor binding motifs (including GC-box/Sp/KLF-type sites), supporting its functional relevance as a critical regulatory segment (Supplementary Table S1).

To investigate whether these observed activity levels correspond to a conserved sequence across species, we aligned the region 2690 bp upstream of the *LGALS3* TSS of selected species with the human *LGALS3* promoter sequence (−2638/+52). The alignment revealed conserved motifs within this upstream region, mainly in primates (positions − 600 to 0 relative to human TSS) and rodents (positions − 2200 to −1200 relative to human TSS), suggesting that this promoter is of evolutionary significance (Supplementary Figures S1 and S2). However, we could not identify conserved regions among other species, e.g., chicken, by using a minimum conservation threshold of 50%.

### Effects of SOX proteins on the activity of the *LGALS3* promoter

We first performed a qPCR analysis to characterize the cell line SW1353 regarding the expression of *SOX* mRNA. As mentioned, SOX9 is a transcription factor dysregulated within OA and could be involved in the regulation of galectins. In addition to SOX9, other SOX family members were included as part of an exploratory analysis. The mRNA levels of the chosen SOX genes (*SOX2*,* SOX4*,* SOX5*,* SOX6*, and *SOX9*) relative to that of GAPDH ranged from 10^− 3^ to 10^− 4^ (Supplementary Figure S3). *SOX2* and *SOX9* were the genes with the lowest relative expression level in the SW1353 cells, yet the Kruskal-Wallis test did not find the difference within the group of SOX genes to be significant.

**Fig. 2:**
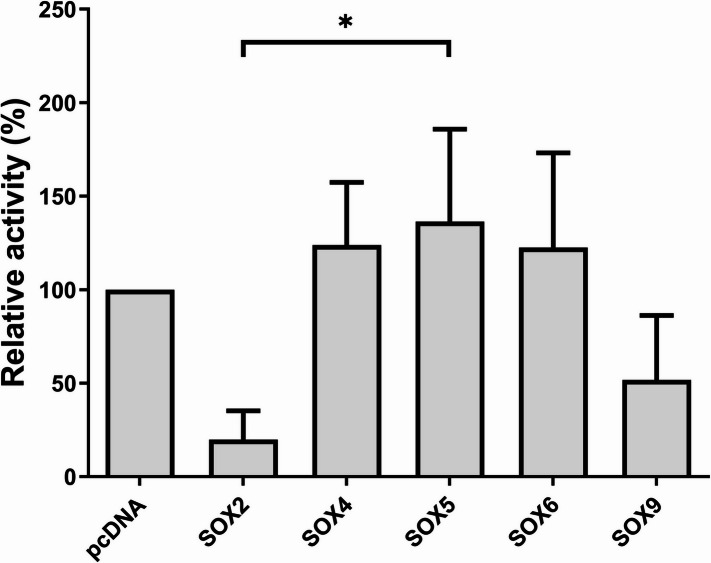
Relative activity of the *LGALS3* full-length promoter (−2638/+52) in SW1353 cells when co-transfected with 30 ng of different SOX-containing plasmids (pcDNA3.1(+)-SOX2, -SOX4, -SOX5, -SOX6 and -SOX9). The relative activity of the promoter in the different conditions is calculated as a ratio to the promoter activity of the cells transfected with the vector pcDNA3.1(+) lacking the respective SOX. The graph shows results’ mean relative activity ± SD from four technical replicates, each including three biological replicates, with error bars representing the standard deviation. *p < 0.05.

Consecutively, by co-transfecting SW1353 cells with 50 ng of a reporter construct containing the full-length *LGALS3* promoter, pGL4-hGal3p(−2638/+52), and 30 ng of plasmids encoding different SOX genes (pcDNA3.1(+)-SOX2, pcDNA3.1(+)-SOX4, pcDNA3.1(+)-SOX5, pcDNA3.1(+)-SOX6, pcDNA3.1(+)-SOX9), we investigated quantitatively their effect on the promoter activity. The vector pcDNA3.1(+), without any SOX encoding sequence, was used as a control. The relative luciferase activities of the full-length promoter in the presence of the SOX proteins were calculated as a percentage referred to the 100% value of the control and are shown in Fig. 2. To confirm that transfections with SOX-encoding plasmids were efficient and the SOX-encoding plasmids caused changes in promoter activity, an additional 24-well plate was processed in the same way and used for qPCR analysis of the SOX genes’ mRNA expression (data not shown). The *SOX2*-containing vector caused repression of *LGALS3* promoter activity by 80.1%, and the *SOX9*-containing vector by 48.3%. As SOX2 is not expressed in cartilage, these findings were not interpreted as biologically relevant but rather used to benchmark potential SOX family effects. In contrast, the *SOX4*-, *SOX5*-, and *SOX6*-containing vectors increased promoter activity by 23.9%, 36.5%, and 22.5%, respectively. The only statistically significant difference found was between *SOX2* and *SOX5* (*p* < 0.05).

### *SOX2* and *SOX9* regulate the *LGALS3* promoter activity in a dose-dependent manner

**Fig. 3:**
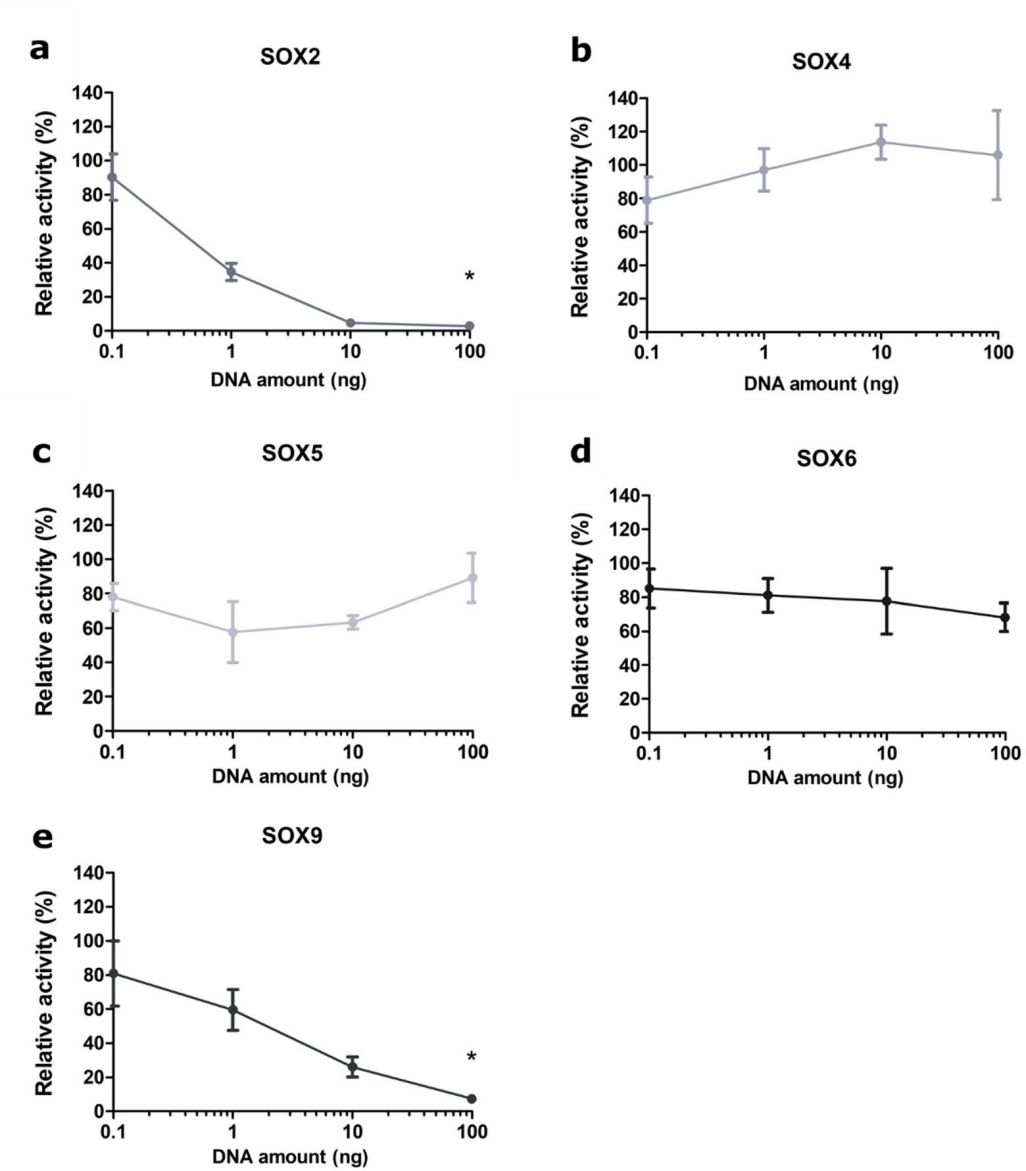
Relative activity of the *LGALS3* full-length promoter (−2638/+52) in SW1353 cells when titrated with different amounts of SOX-containing plasmids. (**a**) pcDNA3.1(+)-SOX2, (**b**) pcDNA3.1(+)-SOX4, (**c**) pcDNA3.1(+)-SOX5, (**d**) pcDNA3.1(+)-SOX6, (**e**) pcDNA3.1(+)-SOX9. The amounts of DNA used were: 0.1 ng, 1 ng, 10 ng, 100 ng. For each condition, a negative control without the SOX-encoding plasmid was included. The x-axis is plotted on a logarithmic scale. The relative activity of the promoter in the different conditions is calculated as a ratio to the promoter activity of the cells transfected with the vector pcDNA3.1(+) lacking the respective SOX. The graph shows results’ mean relative activity ± SD from three biological replicates, including three technical replicates, with error bars representing the standard deviation. *p < 0.05.

To determine dose dependency of *LGALS3* promoter activity by SOX proteins, we repeated the co-transfection experiment mentioned in the previous section with increasing amounts of pcDNA3.1(+)-SOX2, pcDNA3.1(+)-SOX4, pcDNA3.1(+)-SOX5, pcDNA3.1(+)-SOX6 or pcDNA3.1(+)-SOX9 plasmid DNA (0.1, 1, 10, 100 ng). With increasing amounts of *SOX2-* and *SOX9*-encoding plasmids, we observed a stepwise decrease of *LGALS3* promoter activity (Fig. 3). A statistically significant difference in relative activity was detected between samples with the lowest (0.1 ng) and highest (100 ng) plasmid amounts for both *SOX2*- and *SOX9*-encoding constructs (Fig. 3). By transfecting cells with 100 ng of the respective plasmids, we achieved a repression of the *LGALS3* promoter of 97.2% with *SOX2* and 92.6% for *SOX9* (Fig. 3). These findings demonstrate that overexpression of either *SOX2* or *SOX9* alone can suppress *LGALS3* promoter activity in SW1353 cells.

Transfection with varying amounts of pcDNA3.1(+)-SOX4, pcDNA3.1(+)-SOX5, and pcDNA3.1(+)-SOX6 did not result in consistent or dose-dependent changes in *LGALS3* promoter activity. For SOX4, a modest increase was observed at lower plasmid amounts (0.1–10 ng), but this effect was not maintained at 100 ng and was therefore not considered reproducible. SOX5 and SOX6 showed small fluctuations within a narrow activity range (approximately 70–120%), with no systematic trend across the titration series. These variable responses suggest that SOX4, SOX5, and SOX6 do not exert a strong or specific regulatory effect on the *LGALS3* proximal promoter under the tested conditions.

### *In silico* analysis of the *LGALS3* promoter for transcription factor binding sites (TFBS) revealed multiple potential SOX binding sites

**Fig. 4:**
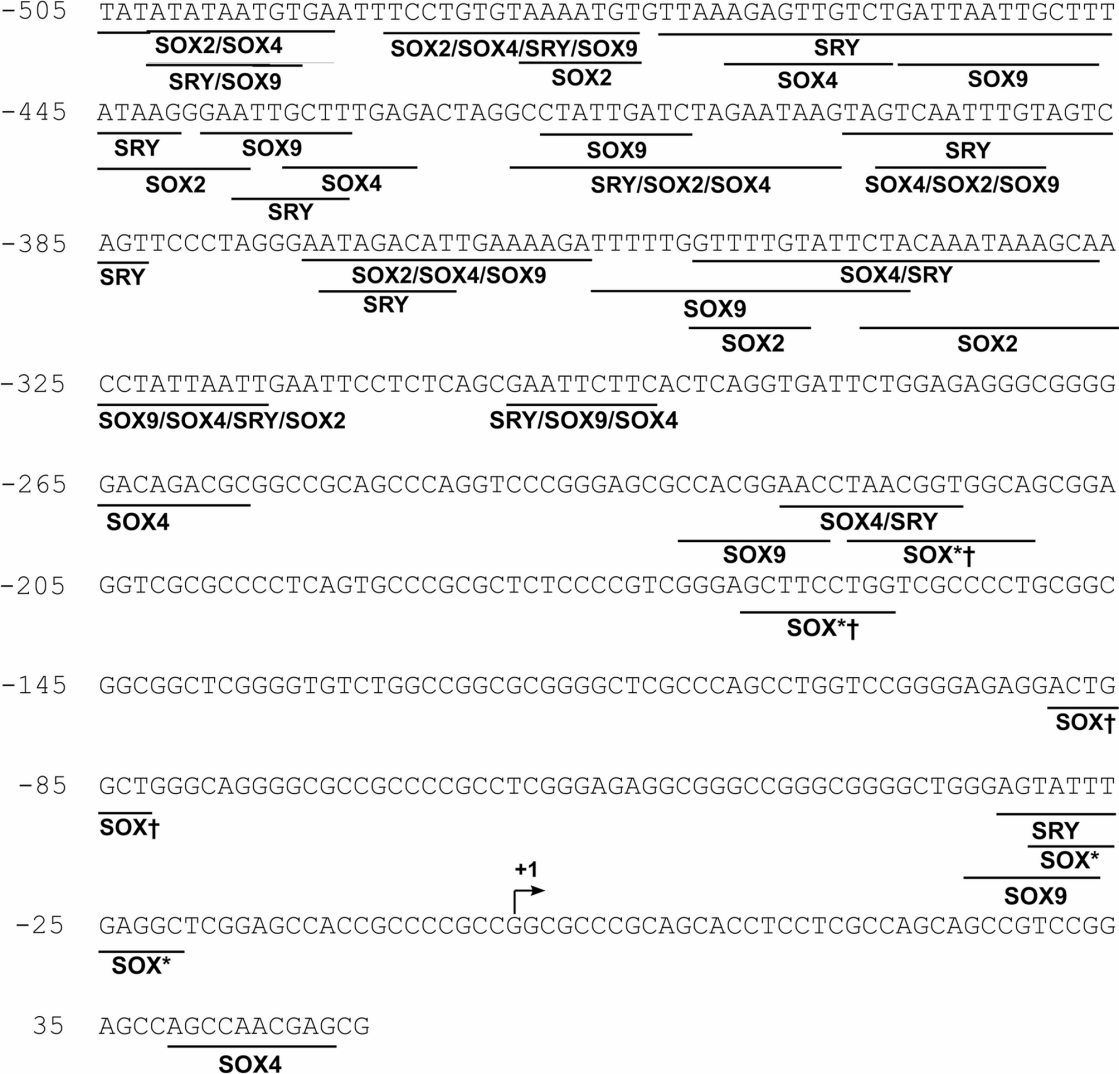
Human *LGALS3* promoter (−505/+52) with potential SOX binding sites. Information on the binding sites was obtained from JASPAR, setting the threshold at 70%. TSS is indicated with an arrow.

An *in silico* analysis using the JASPAR database was performed to find potential SOX-binding sites within the human *LGALS3* promoter region. This analysis aimed to determine whether the observed SOX-mediated effects on *LGALS3* expression could be due to a potential binding within the *LGALS3* promoter sequence. SRY matrices were also included, as SRY and other SOX proteins bind to DNA through their HMG-box domains and are part of the same family: the SOX (SRY-related HMG-box) family. JASPAR predicted 655 SOX-binding sites within the full-length (−2638/+52) *LGALS3* promoter at a threshold of 80%. The 20 results of SOX TBFS with the highest relative score and their relative positions within the promoter sequence are presented in Supplementary Table S2. A region spanning from − 505 to + 52 relative to the TSS is shown with SOX binding sites at a 70% threshold in Fig. 4. We could observe a higher density of SOX TFBS from the − 505 to −325 region than in the − 325 to + 52 region.

SOX9 (Matrix ID MA0077.1.SOX9) could potentially bind to the *LGALS3* promoter on multiple sites, for instance, on the sense strand (+) at positions − 418 to −410 relative to the TSS, with a high relative score (0.92, Fig. 4). For SOX2, multiple putative binding sites were also found (Matrix ID MA0143.5.SOX2) predicted with high relative scores of up to 0.88 (result not shown) to bind to the *LGALS3* promoter on the sense strand (+) at positions − 1462 to −1456 relative to the TSS, and positions − 1133 to −1127 relative to the TSS on the antisense strand (-). SOX4 (Matrix IDs MA0867.3 and MA0867.2.SOX4) binding sites had high relative scores (up to 0.95) at positions − 1833 to −1926 relative to the TSS (Supplementary Table S2).

The analysis also found some SOX proteins that were not the object of this study. However, it is worth mentioning that a binding motif for SOX10 (Matrix IDs MA0442.3 and MA0442.1) was found at position − 1698 to −1693 relative to the TSS, consisting of the complementary sequences 3’-ACAAAG-5’ and 5’-CTTTGT-3’ (Supplementary Table S2). Additionally, we found multiple potential SOX15 (Matrix ID MA1152.2) binding sites (relative scores ranging from 0.93 to 0.98) at various positions, including − 1833 to −1827 and − 2243 to −2237 relative to the TSS (Supplementary Table S5). These potential binding sites suggest that SOX proteins could bind to the *LGALS3* promoter, possibly impacting its regulation. In addition to TFBS for SOX proteins, we found several motifs for other TFs (e.g., KLFs), presented in Supplementary Table S1.

### Effect of *SOX2* and *SOX9* on promoter deletion constructs

**Fig. 5:**
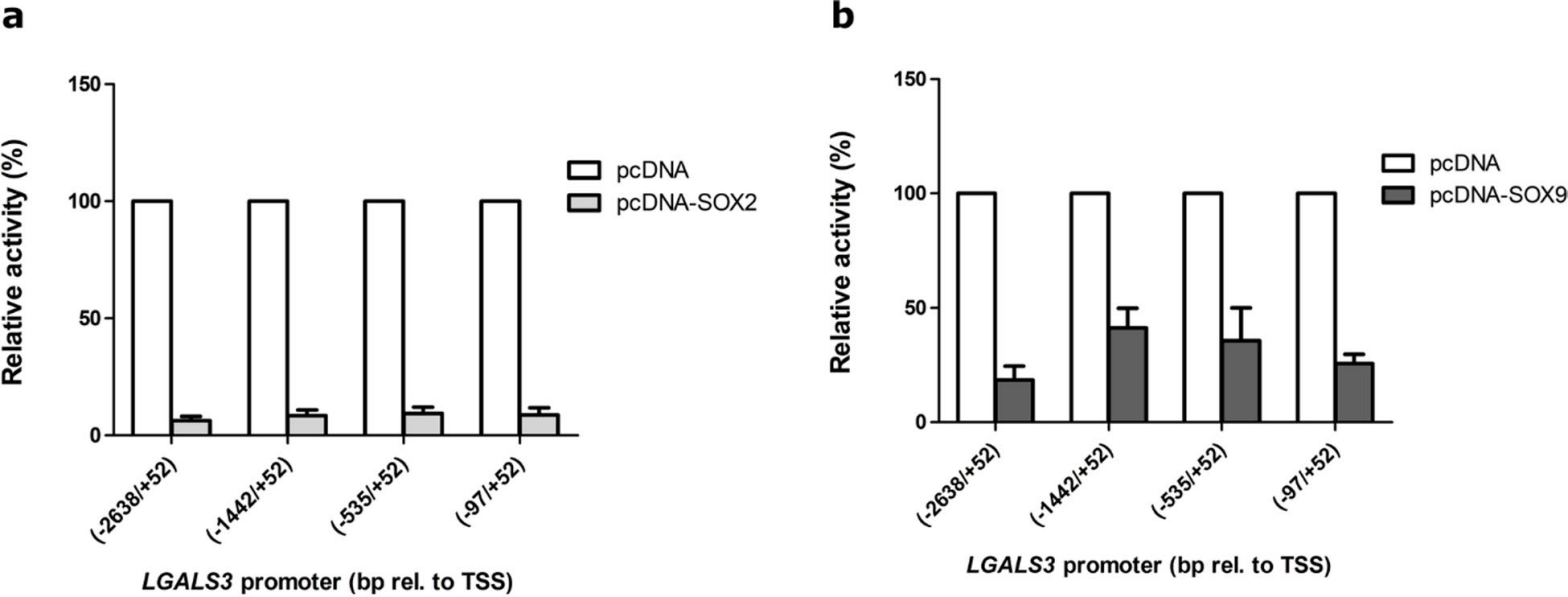
Relative activity of *LGALS3* promoter deletion variants in SW1353 cells when co-transfected for 24 h with SOX-protein-containing plasmids: (**a**) SOX2 or (**b**) SOX9. The graph shows results’ mean ± SD from at least three biological replicates, including three technical replicates, with error bars representing the standard deviation. The white bars represent the negative control (empty pcDNA3.1(+)). (**a**) The light grey bars represent SOX2. (**b**) The dark grey bars represent SOX9.

To explore which sequence stretches are responsible for the SOX2- and SOX9-mediated effects, we examined the impact of pcDNA3.1(+)-SOX2 and pcDNA3.1(+)-SOX9 on *LGALS3* promoter deletion constructs (Fig. 5). Luciferase assays showed that SOX2 exerted similar repression levels on the activity of each deletion variant, with a residual promoter activity between 6.3 ± 1.9% and 9.3 ± 2.8% (Fig. 5a). Since this uniform repression pattern could indicate a squelching effect, we additionally tested SOX2 using an unrelated SV40 promoter. Here, we also observed repression, supporting the notion of a non-specific interference with transcription rather than promoter-dependent regulation (Supplementary Figure S6).

In contrast, SOX9 (Fig. 5b) produced a graded and promoter-length–dependent repression pattern. The strongest reduction was observed for the full-length promoter (−2638/+52), which retained 18.4 ± 6.2% residual activity. Deleting sequences up to −1442 resulted in a partial loss of repression (41.3 ± 8.6%), while shorter constructs again showed lower activities, with the proximal minimal promoter fragment (−97/+52) reaching 25.5 ± 4.0%.

This non-uniform pattern indicates that SOX9 does not act via global squelching but interacts with specific promoter regions. Since repression persisted even in the shortest construct and reached its minimum in the − 97/+52 fragment, these data localize the essential SOX9-responsive element to the proximal 149 bp region upstream and downstream of the transcription start site.

### SOX9 can directly bind to the *LGALS3* promoter

**Fig. 6:**
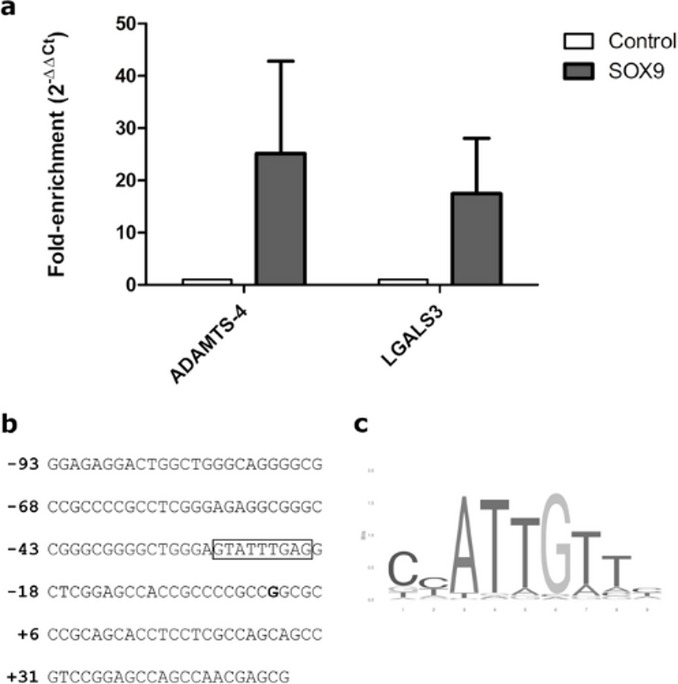
Fold enrichment of specific promoter binding of HaloTag-SOX9 and hGal3p(−93/+49). (**a**) HaloChIP^TM^ experiments were performed using SW1353 cells transfected with HaloTag-SOX9 or with the HaloTag-only vector (pHTN, control). DNA bound to SOX9 was isolated using the HaloCHIP™ system (Promega), and DNA was amplified via quantitative PCR. ADAMTS-4 is a known target of SOX9 and was used as a positive control. The graph shows mean ± SD from two biological replicates, with error bars representing the standard deviation. White bars represent the HaloTag-only control (pHTN). The dark grey bars represent HaloTag-SOX9. (**b**) The sequence stretch studied is shown, with a potential SOX9 binding site shown in a box. (**c**) A canonical SOX9 binding motif is shown.

To assess whether the observed repressive effect of SOX9 on the *LGALS3* promoter activity was due to direct binding of the promoter, we performed a chromatin immunoprecipitation using HaloTag plasmid pHTN-SOX9. We focused on SOX9 because it is a key regulator in cartilage and chondrogenesis, and its transcriptional activity controls numerous cartilage-specific genes and regulatory factors. As a positive control, we used the *ADAMTS*−4 promoter, which is known to interact with the SOX9. In our analysis, SOX9 showed strong binding to the *ADAMTS-4* promoter region, leading to a 25-fold enrichment in our CHIP-qPCR compared to cells transfected with the HaloTag-only vector (pHTN), which served as the negative control in the HaloChIP^TM^ system (control, Fig. 6a). Since the HaloChIP^TM^ system is antibody-independent, enrichment was calculated relative to cells transfected with the HaloTag-only vector lacking SOX9, which controls for nonspecific DNA recovery and HaloTag-mediated capture. For *LGALS3*, we obtained a 17-fold enrichment for the sequence between − 93 and + 49 relative to the TSS with cells transfected with SOX9-HaloTag, proving the binding of SOX9 within this sequence stretch. Figure 6b shows the studied sequence stretch, with a putative SOX9 binding site highlighted, and Fig. 6c shows a canonical SOX9 binding motif.

### Basal *LGALS3* promoter activity is affected by SOX motif mutation

**Fig. 7:**
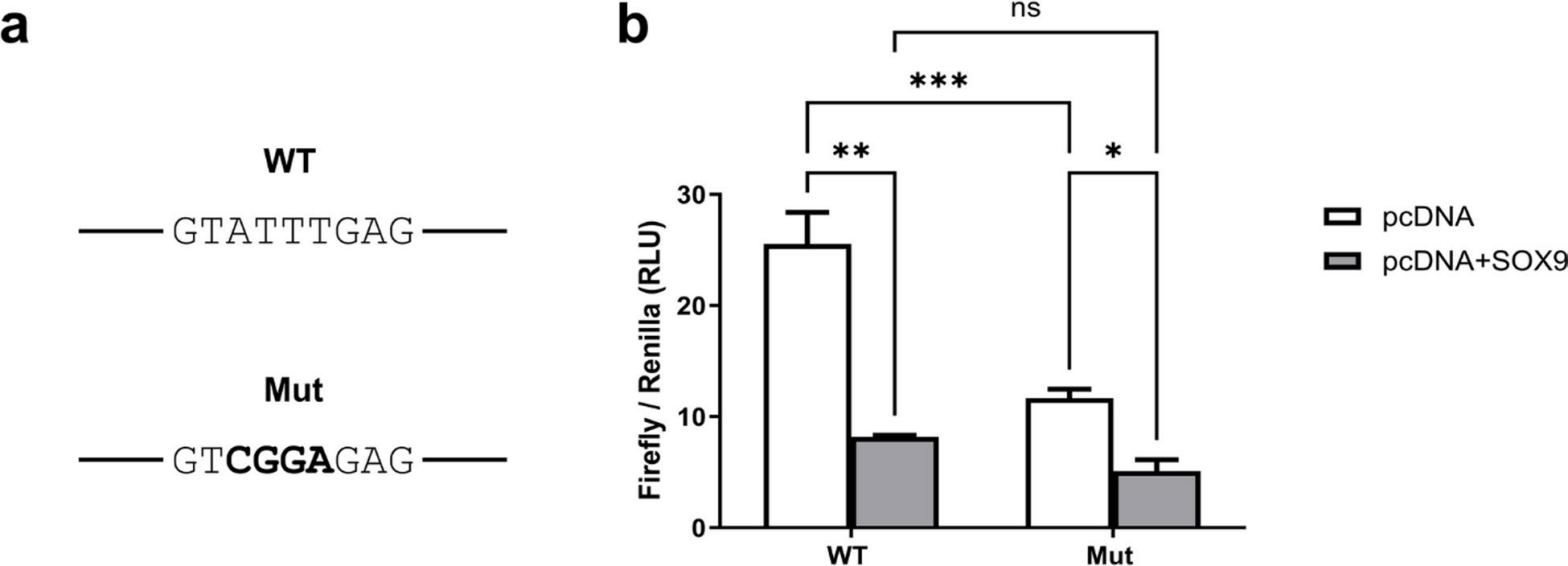
Mutagenesis of the predicted SOX9 binding-site in the *LGALS3* promoter. (**a**) The predicted SOX9 motif is shown within the wild-type sequence (WT); Mut shows the mutated motif. (**b**) Firefly luciferase activity (Firefly/*Renilla* ratio) of the *LGALS3* promoter construct (−97/+52) WT versus the *LGALS3* promoter construct (−97/+52) carrying a mutation in the predicted SOX9 binding site in SW1353 cells, co-transfected with pcDNA (white bars) or pcDNA-*SOX9* (grey bars). Data represent mean ± SD of three independent experiments performed in triplicate. Statistical significance is indicated as *p < 0.05, **p < 0.01, ***p < 0.001.

We next investigated whether the identified SOX9 binding site is functionally required for SOX9-mediated repression of hGal-3p(−97/+52) promoter. Within the enriched sequence stretch, a putative SOX consensus motif (GTATTTGAG) was identified (Fig. 7a). To determine its functional relevance, site-directed mutagenesis was performed within the hGal3p (−97/+52) construct.

Dual luciferase reporter assays were then conducted using wild-type (WT) and mutated (Mut) promoter constructs in the presence or absence of the SOX9 expression vector (Fig. 7b). Mutation of the predicted SOX9 binding site resulted in a significant reduction of basal promoter activity (11.67 ± 1.41) compared to the WT construct (25.55 ± 4.93, *p* = 0.0002), indicating that this region contributes to basal *LGALS3* transcriptional activity. Consistent with our previous observations, SOX9 expression markedly reduced WT promoter activity to 8.2 ± 0.185 (*p* = 0.001). Notably, mutation of the predicted SOX9 binding site did not abolish but slightly lowered SOX9-mediated repression (*p* = 0.0313). Strikingly, robust SOX9-dependent repression was observed in both constructs, corresponding to a 68.18% decrease in WT activity and a 56.48% decrease in the mutant promoter. In conclusion, this promoter region is essential for basal *LGALS3* transcription and partially mediates SOX9-dependent repression, suggesting that additional regulatory elements contribute to SOX9 responsiveness.

### SOX9 lowers *LGALS3* mRNA levels

**Fig. 8:**
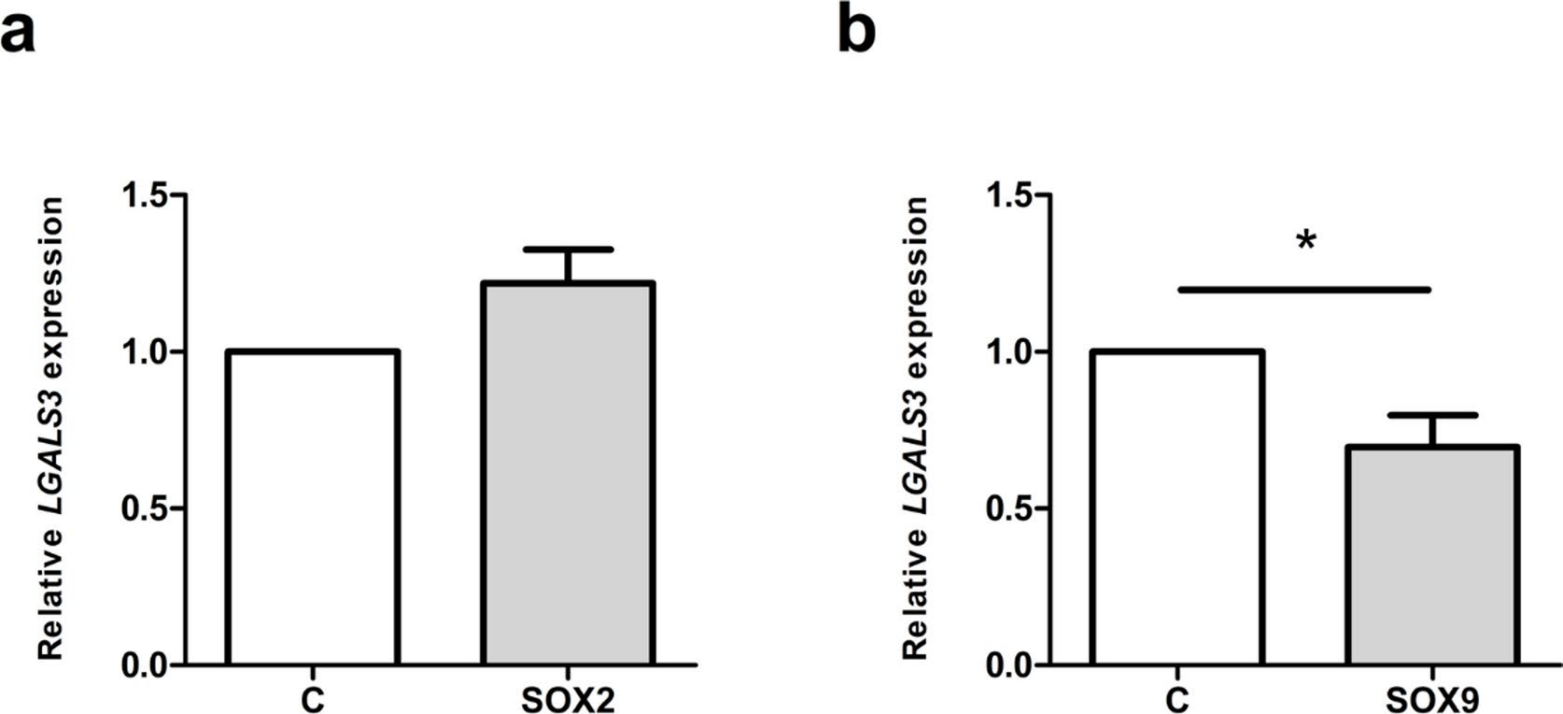
*LGALS3* mRNA levels after overexpression of either (**a**) SOX2 or (**b**) SOX9. The graph shows results’ mean ± SD from three biological replicates, each performed with two technical replicates, with error bars representing the standard deviation. Statistical significance was determined using an unpaired two-tailed Student’s t-test. *p < 0.05.

Finally, to evaluate whether the promoter-based effects translate into changes in endogenous gene expression, we analysed *LGALS3* mRNA levels after SOX2 and SOX9 overexpression. SW1353 were transfected with either SOX2 or SOX9 and *LGALS3* mRNA expression was analysed by qPCR 48 h post transfection. Overexpression of both factors was confirmed by qPCR (Supplementary Figure S7). SOX9 overexpression resulted in a 30% reduction of the *LGALS3* mRNA levels (*p* = 0.0358, Fig. 8b). In contrast, *SOX2* overexpression led to a slight increase in *LGALS3* expression, although this change did not reach statistical significance (*p* = 0.0726, Fig. 8a).

## Discussion

Galectin-3 is a multifunctional β-galactoside-binding lectin implicated in various cellular processes^4,10, 23, 24–39^. Elevated galectin-3 levels occur in several pathological conditions, including OA^10^. We previously reported that its upregulation in OA cartilage is associated with tissue degeneration^10,39^. However, the molecular mechanisms underlying this upregulation remain poorly understood, and the transcriptional regulation of *LGALS3* in chondrocytes has not been explored.

The first aim of this study was to identify sequence regions essential for *LGALS3* promoter activity in SW1353 cells. Deletion constructs revealed that promoter activity remained largely unchanged until the region upstream of −97 was removed, defining a minimal promoter region (−97/+52) of 149 bp. A marked drop-in activity between the − 97/+52 and − 77/+52 constructs indicates that the 20 bp region upstream of − 77 contains a critical activating element. Conversely, the increased activity of the − 1442/+52 construct relative to the longer variants points to distal repressive elements within the − 2638/–1442 region, supporting a modular promoter architecture with both activating and inhibitory sequences. The GC-rich architecture of the minimal promoter and the presence of SP/KLF motifs (Supplementary Table S1) are consistent with regulatory patterns of TATA-less promoters.

SOX proteins are essential regulators throughout chondrogenesis^16,40–42^, whereas in OA, several SOX proteins are dysregulated and contribute to disease progression^14,17,18,43^. The SOX trio (SOX5, SOX6, and SOX9), which is essential for cartilage development and maintenance^16,42^, shows reduced expression in osteoarthritic cartilage, suggesting a loss of chondrocyte identity and function^17^, while SOX4 directly induces expression of catabolic enzymes such as ADAMTS-4 and ADAMTS-5^18^. Likewise, SOX9 has been implicated in regulating cartilage degeneration associated with ADAMTS activity in early OA^43^.

We hypothesised that SOX proteins (SOX2, SOX4, SOX5, SOX6, and SOX9) are involved in regulating the *LGALS3* promoter. We observed that *SOX2* and *SOX9* reduced *LGALS3* promoter activity in a dose-dependent manner (Figs. 2 and 3). In contrast, the effects observed for SOX4, SOX5, and SOX6 were variable, and not reproducible across the titration series. SOX4 showed a mild increase at lower concentrations that did not follow a clear dose-dependent trend, while SOX5 and SOX6 exhibited small fluctuations without a consistent regulatory pattern. For SOX5 and SOX6, the absence of a consistent effect is not unexpected, as these factors are well known to function predominantly as cofactors of SOX9 rather than acting independently. Their individual overexpression therefore does not necessarily lead to a robust regulatory phenotype.

Since *SOX2* and *SOX9* showed the most pronounced effects, we tested the pcDNA3.1(+)-SOX2 and SOX9 constructs with different *LGALS3* promoter deletion variants to narrow the specific regions crucial for repression. SOX2-mediated repression was observed for all promoter constructs, with residual promoter activities between 6.3% and 9.3% (Fig. 5a). The strong repression when tested on an unrelated SV40-driven promoter (pGL4.13) (Supplementary Figure S6) reflects a non-specific transcriptional squelching rather than OA-relevant gene regulation, supporting its lack of biological relevance in OA. SOX9 showed a distinct behaviour. Repression was weaker for the deletion construct (−1442/+52) than for the full-length promoter, which could indicate the presence of a SOX9 TFBS within the − 2638 bp to −1442 bp area, whose deletion led to an increase in the activity. From the (−1442/+52) to the (−97/+52) construct, the activity decreased steadily but not significantly, meaning that activating elements could be removed. Whether these elements include SOX9 TFBS remains unknown. Our findings revealed that SOX9 could transcriptionally repress *LGALS3* expression. Remarkably, SOX9 - a key regulator of chondrogenesis - is downregulated in OA cartilage^44^, while we showed that galectin-3 expression in OA cartilage is increased^10^. The reduced SOX9 expression in OA chondrocytes may relieve repression of the *LGALS3* promoter, potentially contributing to the observed upregulation of galectin-3 in OA^10,39^.

Additional evidence suggests that galectins and SOX proteins may participate in broader regulatory feedback loops. Galectin-1 has been reported to influence SOX9 expression in colorectal cancer models^19^, and galectin-3 can modulate SOX2 levels during osteogenic differentiation^45^. Song et al. (2005)^35^ showed that galectin-3 could form a complex with AP-1 and DNA, as demonstrated by chromatin immunoprecipitation and pull-down. Since AP-1 is also known to interact with SOX9^46^, this further supports the notion that SOX proteins may participate in multi-factorial complexes influencing galectin regulation. Zhang et al. (2015)^43^ reported SOX9 as an activator and repressor, describing that “SOX9 repressed ADAMTS’ expression and promoted COL2A1, ACAN, and COMP expression in human chondrocytes”. Our findings indicate that SOX9 represses *LGALS3* promoter activity, and this is substantiated by our HALO-ChIP data showing direct SOX9 binding within the − 93/+49 region of the *LGALS3* promoter, with a 17-fold enrichment compared to the HaloTag-only vector (pHTN, control). This result is the first proof of direct SOX9 binding to the *LGALS3* promoter, identifying a potentially critical regulatory mechanism for Gal-3 dysregulation in OA.

However, despite direct binding to the promoter, we cannot exclude that SOX9 may also repress the *LGALS3* promoter through other mechanisms, such as inhibition of the Wnt/β-catenin signalling pathway. TFBS analysis using JASPAR identified several high-scoring TCF binding sites in the *LGALS3* promoter (with a 70% threshold), indicating a potential role for regulation through the Wnt/β-catenin signalling. Supplementary Figure S4 shows several TCF binding sites distributed across the (−505/+52) region. SOX9 possesses an inhibitory activity through the Wnt/β-catenin pathway^41,47^ by direct competition with TCF/Lef for β-catenin binding, leading to its degradation^47,48^. We could hypothesise that this competitive binding is responsible for SOX9-mediated repression of *LGALS3* expression. Thus, the inhibitory effect of SOX9 on *LGALS3* transcription could result from direct promoter binding or indirect regulation via β-catenin degradation, potentially involving Wnt signalling.

Our next aim was to investigate whether the predicted SOX consensus motif (GTATTTGAG) within the Gal-3p(−97/+52) promoter is required for SOX9-mediated repression. Site-directed mutagenesis of this motif markedly reduced basal promoter activity, indicating that it represents an important activating element of the *LGALS3* promoter. Interestingly, the mutant promoter showed slightly lower repression by SOX9, therefore it is likely that either additional motifs or indirect mechanisms contribute to SOX9 responsiveness.

To investigate whether repression translates to endogenous gene regulation, we examined *LGALS3* mRNA levels following SOX overexpression in SW1353 cells. SOX9 significantly reduced *LGALS3* mRNA, consistent with its repressive effect in luciferase assays. In contrast SOX2 did not decrease *LGALS3* transcript levels despite strongly repressing promoter activity in reporter assays.

The weaker effects on endogenous *LGALS3* mRNA compared to the strong repression observed in luciferase assays can be explained by several factors. Luciferase reporters capture promoter regulation in an isolated, out-of-genome context, whereas the endogenous *LGALS3* locus is embedded within a complex chromatin environment containing additional regulatory elements that may buffer or counteract transcriptional changes. Moreover, post-transcriptional mechanisms, such as mRNA stabilisation, may maintain *LGALS3* transcript levels despite reduced promoter output.

For SOX2 in particular, the discrepancy is further explained by its squelching-based, non-specific effect in reporter assays, which does not translate to physiological regulation of the endogenous gene. In contrast, SOX9 shows consistent repression at both the promoter and transcript levels, although the magnitude of mRNA reduction is modest relative to its strong promoter repression. This suggests that additional promoter-proximal or distal elements, cofactors, or chromatin states modulate the ultimate transcriptional output of *LGALS3* in SW1353 cells.

Since our overall aim was to investigate the regulation of *LGALS3* in OA, the question arose as to what extent the SW1353 chondrosarcoma cells can be a model for OA chondrocytes. When comparing mRNA expression levels of *LGALS3* and the SOX transcription factors of interest in OA primary chondrocytes (Supplementary Figure S5a with those in the SW1353 cells (Supplementary Figure S5b), we observed that *LGALS3*,* SOX4*,* SOX5* and *SOX9* were expressed at higher levels than in SW1353 cells. Thus, although SW1353 cells allowed us to define core regulatory principles of *LGALS3* promoter control, their use as an OA model is inherently limited. As a cancer-derived cell line, SW1353 cells likely have epigenetic alterations that may affect chromatin accessibility at the *LGALS3* promoter. Consequently, findings obtained in SW1353 cells cannot be directly extrapolated to OA chondrocytes.

Future studies should investigate these mechanisms in OA primary chondrocytes and, ideally, in relevant *in vivo* models to validate whether SOX proteins directly control *LGALS3* in the OA context. Such work will be essential to determine whether the promoter-level interactions identified here translate into changes in endogenous *LGALS3* expression, chondrocyte phenotype, or OA-associated cellular responses. Dedicated loss- and gain-of-function experiments in primary cells and animal models will be required to establish whether SOX-dependent pathways converge on *LGALS3* and whether this regulatory axis contributes to disease progression *in vivo*.

Understanding how SOX factors influence *LGALS3* expression may also provide conceptual entry points for therapeutic modulation. Since Gal-3 promotes inflammatory and degenerative processes in OA, reducing its expression through manipulation of upstream regulatory networks could be beneficial. Although direct targeting of transcription factors such as SOX9 remains challenging, emerging strategies—including epigenetic modulation, enhancer-targeting approaches, and RNA-based therapeutics—may offer future avenues to fine-tune *LGALS3* expression. Defining the regulatory circuitry of *LGALS3* therefore establishes a foundational framework for mechanistic validation and therapeutic exploration in OA.

In conclusion, we identified the minimal active promoter sequence of human *LGALS3* and characterised the regulatory impact of *SOX2*, *SOX4*, *SOX5*, *SOX6*, and *SOX9* on its activity.

We observed stable promoter activity until the region − 2638/−97 was deleted, highlighting the importance of the − 97/+52 region. Among the SOX factors tested, SOX9 appeared to be a repressor of the human *LGALS3* promoter and interacts with the − 97/+52 region. These findings define a core regulatory mechanism controlling *LGALS3* expression and provide a framework for future studies investigating galectin regulation in OA and other inflammation-associated diseases.

## Materials and methods

### Chondrocyte cell culture

SW1353 human chondrosarcoma cells (ATCC HTB-94™, American Type Culture Collection (ATCC)) were cultured at 37 °C in a humidified atmosphere with 5% CO_2_ in Dulbecco’s Modified Eagles Medium (DMEM), containing 10% FCS and 1% penicillin/streptomycin/L-glutamine. Cells were obtained from ATCC and used according to the supplier’s recommendations. Primary OA chondrocytes were isolated from human articular knee joint cartilage obtained from OA patients during total knee replacement surgery with written informed consent and following the terms of the ethics committee of the Medical University of Vienna (EK-No. 1822/2017 and 1555/2019). All methods were carried out in accordance with the approved guidelines and the Declaration of Helsinki. Primary chondrocytes were cultured under standard conditions^49^ and used without subculturing to preserve the chondrocyte phenotype.

### Plasmid extraction

Plasmid extraction was performed with GeneJET Plasmid-MiniPrep-Kit (Thermo Fisher Scientific, Dreieich, Germany) according to the manufacturer’s instructions. The purified plasmid was eluted in 50 µL of water to be able to use the plasmid for sequencing and further downstream applications. The plasmids were obtained from *E. coli* TOP10 chemically competent cells (Thermo Fisher Scientific). The plasmids used are listed in Supplementary Table S3.

### RNA isolation and quantitative PCR (qPCR) analysis

Total RNA was isolated from primary OA chondrocytes and SW1353 cells using the Invitrogen™ RNA Mini Kit (Thermo Fisher Scientific) following the manufacturer’s instructions, adding a DNase digestion step to avoid genomic DNA contamination. For RNA extraction from transfected cells, the SW1353 cells were cultured in a 6-well plate and harvested 48 h post-transfection. For cDNA synthesis, 2 µg of the isolated RNA was reverse transcribed using the GoScript™ Reverse Transcription System (Promega, Walldorf, Germany). RNA concentration and purity were assessed using a Nanophotometer IMPLEN^®^ P300 (IMPLEN, Munich, Germany), with A260/A280 ratios between 1.98 and 2.07 indicating high purity. RNA integrity was evaluated by agarose gel electrophoresis, confirming the presence of intact rRNA bands. For qPCR, we used 10 µL of LUNA^®^ Universal qPCR Master Mix (NEB, Frankfurt am Main, Germany) in a final reaction volume of 20 µL. Reactions were carried out in technical triplicates on the CFX Duet Real-Time PCR System (Bio-Rad, Munich, Germany), and data were processed using the Bio-Rad CFX Maestro software. Real-time PCR primers were designed manually, checking for primer-dimer formation and secondary structures using the OligoEvaluator™ tool (https://www.oligoevaluator.com/LoginServlet). Primer sequences are presented in Supplementary Table S4. Primers were designed to span exon-exon junctions, to minimize genomic DNA amplification when possible. Amplification efficiencies were determined for each primer pair from standard curves using serial dilutions of cDNA, and only primer sets with efficiencies between 106 and 112% and R² values close to 1 (Supplementary Table S5) were included in the analysis. Melt curve analysis confirmed a single peak, meaning specific amplification products.

### Construction of human *LGALS3* promoter reporter gene vectors

The promoter sequence was amplified from the genomic DNA of SW1353 cells. We used the Wizard^®^ Genomic DNA purification kit (Promega) to extract the genomic DNA. We amplified the first fragment of the promoter (hGal3p-I) by using Expand™ Long Template PCR-System (Roche, Mannheim, Germany). The second fragment (hGal3p-II) was amplified by GC-RICH PCR-System (Roche). Both fragments were first subcloned into pGEM-T Easy (Promega), then digested with *NheI* and *EcoRI* (hGal3p-I) or *EcoRI* and *HindIII* (hGal3p-II). The resulting fragments were then ligated in a three-fragment ligation into pGL4.20 that was cut using *NheI* and *HindIII* and dephosphorylated using the rAPid Alkaline Phosphatase (Roche) to create the pGL4-hGal3p(−2636/+52) reporter vector. Sequence deletions of the *LGALS3* promoter were cloned accordingly using GC-RICH PCR-System (Roche) or Phusion^®^ High-Fidelity DNA Polymerase (NEB). Primer sequences used are in Supplementary Table S4. The sequences of the constructs were all verified by commercial sequencing.

### Construction of transcription factor expression vectors

The coding sequences of the human transcription factors *SOX2*, *SOX4*,* SOX5*, and *SOX6* were amplified by PCR from cDNA of HEK293 cells. The sequence of *SOX9* was amplified from a commercial plasmid, *FUW-tetO-SOX9*, which was a gift from Lorenz Studer (Addgene, Watertown, US; plasmid #141404; RRID: Addgene 141404)^50^ using the primer sets listed in Supplementary Table S4. The amplicons were digested with the appropriate restriction enzymes and directly ligated into the expression vector pcDNA^TM^3.1(+) (Thermo Fisher Scientific). Plasmids were commercially sequenced to verify the inserted sequences.

### Site-directed mutagenesis

QuikChange™ site-directed mutagenesis PCR^51^ was used to insert a transversion mutation in the SOX9 binding site found in the (−97/+52) *LGALS3* promoter-construct. Primers were designed targeting this binding site, presented on Supplementary Table S4. PfuTurbo DNA polymerase (Agilent, Waldbrunn, Germany) and 3% DMSO were used in the PCR reaction.

### Cell transfection

For transfection, SW1353 cells were grown until 70–80% confluency. A volume of 0.5 mL of a 2 × 10^5^ cells/mL cell suspension was seeded into each well of a 24-well cell culture plate. Alternatively, 2 mL of this cell suspension were seeded in the experiments using 6-well plates. After incubation for 24 h, the cells in 24-well plates were transfected with 500 ng of DNA per well using the PEI STAR™ (Tocris Bioscience™, Bristol, UK) transfection reagent in a ratio of 2 µL of PEI STAR™ (1 mg/mL) per 1 µg of DNA according to the manufacturer’s protocol. Similarly, the cells in 6-well plates were transfected with 2 µg of DNA following the same PEI STAR™ (Tocris Bioscience™) ratio.

The transfection mixtures for luciferase reporter assays contain 50 ng of pGL4-hGal-3 promoter plasmid, 10 ng pGL4.74 (Renilla luciferase) for normalization; for the overexpression experiments, increasing amounts (ranging from 0.1 to 100 ng) of the transcription factor-expressing pcDNA3.1(+) plasmid; and for testing unspecific SOX2 effects, we used the unrelated SV40 promoter vector (pGL4.13[luc2/SV40], Promega). Each mixture was supplemented with an empty pGEM^®^-T Easy vector (Promega) to reach 500 ng DNA per 24-well in all transfection tubes, ensuring equal DNA amounts. The culture plate was gently agitated, and the cells were grown for 24 h before further processing, adding 500 µL of media after 2 h to avoid toxicity from the transfection reagent.

### Luciferase reporter gene assays

The transfected SW1353 cells were washed once with PBS after discarding the medium. The cells were lysed using 1 x Passive Lysis Buffer (PLB) from the Dual-Luciferase^®^ Reporter Assay System (Promega). To 20 µl of cell lysate in a white 96-well plate, we added sequentially 50 µl of the firefly and *Renilla* substrate. The plate reader Infinite 200 (Tecan, Crailsheim, Germany) quantified the signal with an integration time of 5 s for each reaction. The signal ratio of firefly/*Renilla* was calculated to ensure normalization of the values.

### Sequence alignment (mVISTA and Jalview software)

For the sequence alignments, we searched for the *LGALS3* orthologues in a selection of species representing different taxonomic groups: Rhesus monkey (*Macaca mulatta*, Gene ID: 697290), orang-utan (*Pongo abelii*, Gene ID: 100453870), mouse (*Mus musculus*, Gene ID: 16854), rat (*Rattus norvegicus*, Gene ID: 83781), dog (*Canis lupus familiaris*, Gene ID: 404021), chicken (*Gallus gallus*, Gene ID: 373917), pig (*Sus scrofa*, Gene ID: 100038033), horse (*Equus ferus caballus*, Gene ID: 100050367) and zebrafish (*Danio rerio*, Gene ID: 557373). Subsequently, all sequences were standardized to a length of 2690 bp upstream the translational start ATG and aligned to the 2690 bp long human sequence (*Homo sapiens*, Gene ID: 3958) using the mVISTA software^52,53^(available at https://genome.lbl.gov/vista/mvista/submit.shtml). For a more detailed analysis, we performed a multiple sequence alignment of the − 97 to +52 region in Jalview (version 2.11.4.1) using the MAFFT algorithm, and results were viewed using the same software.

### Computational analyses

JASPAR software (10th release (2024) https://jaspar.elixir.no)^54^, with a percentage threshold indicated in the different analyses, was used to identify potential transcription factor-binding sites. qPCR data were analysed using the ∆∆Ct method. The ∆∆Ct method was applied for comparing gene expression between the SW1353 cell line and the OA chondrocytes to characterise their *SOX2*,* SOX4*,* SOX5*,* SOX6*,* SOX9*, *and LGALS3* expression profiles. To calculate ΔCt, we normalised the Ct values of the target genes to those of GAPDH. For ΔΔCt values, the ΔCt values of the target genes in primary OA chondrocytes were related to those in the SW1353 cells.

### HaloCHIP™-chromatin immunoprecipitation

We used the pcDNA^TM^3.1(+)-SOX9 vector as a template to clone *SOX9* into the pHTN vector (Promega), resulting in the plasmid pHTN-SOX9, which encodes an N-terminal fusion of the Halo-Tag (33 kDa) with the human SOX9 protein. For transfection the SW1353 cells were seeded in a 6-well plate. After cells reaching a density of 80%, they were transfected with 1.5 µg of pHTN-SOX9 using 3 µl of PEI STAR™ transfection reagent. Further processing was done as described previously^55^. As a negative control, cells were transfected with the pHTN vector encoding HaloTag without SOX9. This control accounts for nonspecific chromatin capture and HaloTag-mediated background binding.

### Statistical procedures

Statistical comparisons between groups were performed using GraphPad Prism software version 10.0.2 (GraphPad Software, Boston, MA, USA). For experiments involving more than two independent groups, the non-parametric Kruskal-Wallis test followed by Dunn’s multiple comparisons test was used. For the SOX9 promoter mutagenesis reporter assays, normalised Firefly/Renilla ratio were analyzed using a two-way ANOVA with the factors *Promoter* (WT vs. Mut) and *SOX9 treatment* (pcDNA vs. SOX9). The analysis included tests for main effects and the interaction between both factors. Post-hoc comparisons between the four relevant conditions (WT ± SOX9; Mut ± SOX9) were performed using Fisher’s LSD tests, as implemented by GraphPad Prism for 2 × 2 designs. Results were considered statistically significant at *p < 0.05, **p < 0.01, and ***p < 0.001.

## Supplementary Information

Below is the link to the electronic supplementary material.

Supplementary Material 1

## Data Availability

The gene and promoter sequences used and analysed during the current study are publicly available in the NCBI repository. For the *LGALS3* promoter (Gene ID: 3958) we used the region between 55.126.601 to 55.129.301. The NCBI RefSeq transcript identifiers for the SOX transcription factors used in this study are as follows: SOX2 (NM\_003106.4), SOX4 (NM\_003107.3), SOX5 (NM\_006940.6), SOX6 (NM\_001145819.2) and SOX9 (NM\_000346.4). The datasets generated and analysed during the current study are available in the figshare repository: [https://figshare.com/articles/dataset/Raw\_data\_for\_Figures/29821859](https:/figshare.com/articles/dataset/Raw_data_for_Figures/29821859).

## References

[1] Barondes, S. H., Cooper, D. N., Gitt, M. A. & Leffler, H. Galectins. Structure and function of a large family of animal lectins. *J. Biol. Chem.* **269**, 20807–20810. 10.1016/s0021-9258(17)31891-4 (1994). 8063692

[2] Johannes, L., Jacob, R. & Leffler, H. Galectins at a glance. *J. Cell Sci.*10.1242/jcs.208884 (2018). 29717004 10.1242/jcs.208884

[3] Stowell, S. R. et al. (eds) *Galectins* Vol. 2442 (2022).

[4] Issa, S. F. et al. Increased galectin-3 may serve as a serologic signature of pre-rheumatoid arthritis while markers of synovitis and cartilage do not differ between early undifferentiated arthritis subsets. *Arthritis Res. Ther.* **19**, 80. 10.1186/s13075-017-1282-4 (2017). 28446218 10.1186/s13075-017-1282-4 PMC5407000

[5] Ohshima, S. et al. Galectin 3 and its binding protein in rheumatoid arthritis. *Arthritis Rheum.* **48**, 2788–2795. 10.1002/art.11287 (2003). 14558084 10.1002/art.11287

[6] Li, S., Yu, Y., Koehn, C. D., Zhang, Z. & Su, K. Galectins in the pathogenesis of rheumatoid arthritis. *J. Clin. Cell. Immunol.*10.4172/2155-9899.1000164 (2013). 24416634 10.4172/2155-9899.1000164 PMC3886720

[7] Gullo, T. R. et al. Defining multiple joint osteoarthritis, its frequency and impact in a community-based cohort. *Semin. Arthritis Rheum.* **48**, 950–957. 10.1016/j.semarthrit.2018.10.001 (2019). 30390991 10.1016/j.semarthrit.2018.10.001 PMC6456431

[8] Hawker, G. A. Osteoarthritis is a serious disease. *Clin. Exp. Rheumatol.* (2019). 31621562

[9] Kraus, V. B., Blanco, F. J., Englund, M., Karsdal, M. A. & Lohmander, L. Call for standardized definitions of osteoarthritis and risk stratification for clinical trials and clinical use. *Osteoarthr. Cartil.* **23**, 1233–1241. 10.1016/j.joca.2015.03.036 (2015). 10.1016/j.joca.2015.03.036 PMC4516635 25865392

[10] Weinmann, D. et al. Galectin-3 induces a pro-degradative/inflammatory gene signature in human chondrocytes, teaming up with Galectin-1 in osteoarthritis pathogenesis. *Sci. Rep.* **6**, 39112. 10.1038/srep39112 (2016). 27982117 10.1038/srep39112 PMC5159921

[11] Pichler, K. M. et al. The dysregulated galectin network activates NF-κb to induce disease markers and matrix degeneration in 3D pellet cultures of osteoarthritic chondrocytes. *Calcif. Tissue Int.* **108**, 377–390. 10.1007/s00223-020-00774-4 (2021). 33185768 10.1007/s00223-020-00774-4 PMC7881967

[12] Li, Z. et al. Activation of Galectin-3 (*LGALS3*) transcription by injurious stimuli in the liver is commonly mediated by BRG1. *Front. Cell Dev. Biol.* **7**, 310. 10.3389/fcell.2019.00310 (2019). 31850346 10.3389/fcell.2019.00310 PMC6901944

[13] Ruebel, K. H. et al. Effects of DNA methylation on Galectin-3 expression in pituitary tumors. *Cancer Res.* **65**, 1136–1140. 10.1158/0008-5472.CAN-04-3578 (2005). 15734994 10.1158/0008-5472.CAN-04-3578

[14] Fisch, K. M. et al. Identification of transcription factors responsible for dysregulated networks in human osteoarthritis cartilage by global gene expression analysis. *Osteoarthritis Cartilage* **26**, 1531–1538. 10.1016/j.joca.2018.07.012 (2018). 30081074 10.1016/j.joca.2018.07.012 PMC6245598

[15] Wegner, M. From head to toes: The multiple facets of Sox proteins. *Nucleic Acids Res.* **27**, 1409–1420. 10.1093/nar/27.6.1409 (1999). 10037800 10.1093/nar/27.6.1409 PMC148332

[16] Ikeda, T. et al. The combination of SOX5, SOX6, and SOX9 (the SOX Trio) provides signals sufficient for induction of permanent cartilage. *Arthritis Rheum.*10.1002/art.20611 (2004). 15529345 10.1002/art.20611

[17] Lee, J. S. & Im, G. I. SOX trio decrease in the articular cartilage with the advancement of osteoarthritis. *Connect. Tissue Res.* **52**, 496–502. 10.3109/03008207.2011.585409 (2011). 21728837 10.3109/03008207.2011.585409

[18] Takahata, Y. et al. Sox4 is involved in osteoarthritic cartilage deterioration through induction of ADAMTS4 and ADAMTS5. *FASEB J.* **33**, 619–630. 10.1096/fj.201800259R (2019). 30016600 10.1096/fj.201800259R

[19] Peng, K. Y. et al. Stromal Galectin-1 promotes colorectal cancer cancer-initiating cell features and disease dissemination through SOX9 and β-Catenin: Development of niche-based biomarkers. *Front. Oncol.* **11**, 716055. 10.3389/fonc.2021.716055 (2021). 34568045 10.3389/fonc.2021.716055 PMC8462299

[20] Schaefer, J. F., Millham, M. L., de Crombrugghe, B. & Buckbinder, L. FGF signaling antagonizes cytokine-mediated repression of Sox9 in SW1353 chondrosarcoma cells. *Osteoarthritis Cartilage* **11**, 233–241. 10.1016/s1063-4584(02)00354-0 (2003). 12681949 10.1016/s1063-4584(02)00354-0

[21] Sang, W. et al. METTL3 involves the progression of osteoarthritis probably by affecting ECM degradation and regulating the inflammatory response. *Life Sci.* **278**, 119528. 10.1016/j.lfs.2021.119528 (2021). 33894271 10.1016/j.lfs.2021.119528

[22] Lila, A. M., Gromova, O. A., Torshin, I. Y. & Montell, E. Molecular effects of chondroitin sulfate in osteoarthritis and herniated discs. *Journal of Rheumatology and Arthritic Diseases* **3**, 1–11. 10.15226/2475-4676/3/3/00143 (2018).

[23] Shimura, T. et al. Galectin-3, a novel binding partner of beta-catenin. *Cancer Res.* **64**, 6363–6367. 10.1158/0008-5472.CAN-04-1816 (2004). 15374939 10.1158/0008-5472.CAN-04-1816

[24] Ahmed, R., Anam, K. & Ahmed, H. Development of Galectin-3 targeting drugs for therapeutic applications in various diseases. *Int. J. Mol. Sci.*10.3390/ijms24098116 (2023). 37175823 10.3390/ijms24098116 PMC10179732

[25] Bouffette, S., Botez, I. & De Ceuninck, F. Targeting galectin-3 in inflammatory and fibrotic diseases. *Trends Pharmacol. Sci.* **44**, 519–531. 10.1016/j.tips.2023.06.001 (2023). 37391294 10.1016/j.tips.2023.06.001

[26] Cervantes-Alvarez, E. et al. Galectin-3 as a potential prognostic biomarker of severe COVID-19 in SARS-CoV-2 infected patients. *Sci. Rep.* **12**, 1856. 10.1038/s41598-022-05968-4 (2022). 35115644 10.1038/s41598-022-05968-4 PMC8813958

[27] Chen, X. et al. Intracellular galectin-3 is a lipopolysaccharide sensor that promotes glycolysis through mTORC1 activation. *Nat. Commun.* **13**, 7578. 10.1038/s41467-022-35334-x (2022). 36481721 10.1038/s41467-022-35334-x PMC9732310

[28] Curti, B. D. et al. Enhancing clinical and immunological effects of anti-PD-1 with belapectin, a galectin-3 inhibitor. *J. Immunother. Cancer*10.1136/jitc-2021-002371 (2021). 33837055 10.1136/jitc-2021-002371 PMC8043038

[29] Hara, A. et al. Galectin-3 as a next-generation biomarker for detecting early stage of various diseases. *Biomolecules*10.3390/biom10030389 (2020). 32138174 10.3390/biom10030389 PMC7175224

[30] Jia, W., Wang, Z., Gao, C., Wu, J. & Wu, Q. Trajectory modeling of endothelial-to-mesenchymal transition reveals galectin-3 as a mediator in pulmonary fibrosis. *Cell Death Dis.* **12**, 327. 10.1038/s41419-021-03603-0 (2021). 33771973 10.1038/s41419-021-03603-0 PMC7998015

[31] Li, Y., Li, T., Zhou, Z. & Xiao, Y. Emerging roles of galectin-3 in diabetes and diabetes complications: A snapshot. *Rev. Endocr. Metab. Disord.* **23**, 569–577. 10.1007/s11154-021-09704-7 (2022). 35083706 10.1007/s11154-021-09704-7 PMC9156459

[32] Liu, Y. et al. Galectin-3 regulates microglial activation and promotes inflammation through TLR4/MyD88/NF-kB in experimental autoimmune uveitis. *Clin. Immunol.* **236**, 108939. 10.1016/j.clim.2022.108939 (2022). 35121106 10.1016/j.clim.2022.108939

[33] Lo, T. H. et al. Galectin-3 promotes noncanonical inflammasome activation through intracellular binding to lipopolysaccharide glycans. *Proc. Natl. Acad. Sci. U. S. A.*10.1073/pnas.2026246118 (2021). 34301890 10.1073/pnas.2026246118 PMC8325309

[34] Pan, X. et al. Pectic polysaccharide from *Smilax china* L. ameliorated ulcerative colitis by inhibiting the galectin-3/NLRP3 inflammasome pathway. *Carbohydr. Polym.* **277**, 118864. 10.1016/j.carbpol.2021.118864 (2022). 34893269 10.1016/j.carbpol.2021.118864

[35] Song, S. et al. Galectin-3 modulates MUC2 mucin expression in human colon cancer cells at the level of transcription via AP-1 activation. *Gastroenterology* **129**, 1581–1591. 10.1053/j.gastro.2005.09.002 (2005). 16285957 10.1053/j.gastro.2005.09.002

[36] Sturgill, E. R. et al. Galectin-3 inhibition with belapectin combined with anti-OX40 therapy reprograms the tumor microenvironment to favor anti-tumor immunity. *Oncoimmunology* **10**, 1892265. 10.1080/2162402X.2021.1892265 (2021). 33717655 10.1080/2162402X.2021.1892265 PMC7927986

[37] Wang, Z. et al. Melatonin inhibits atherosclerosis progression via galectin-3 downregulation to enhance autophagy and inhibit inflammation. *J. Pineal Res.* **74**, e12855. 10.1111/jpi.12855 (2023). 36692032 10.1111/jpi.12855

[38] Zaborska, B. et al. The role of Galectin-3 in heart failure-the diagnostic, prognostic and therapeutic potential-where do we stand?. *Int. J. Mol. Sci.*10.3390/ijms241713111 (2023). 37685918 10.3390/ijms241713111 PMC10488150

[39] Toegel, S. et al. Human osteoarthritic knee cartilage: Fingerprinting of adhesion/growth-regulatory galectins in vitro and in situ indicates differential upregulation in severe degeneration. *Histochem. Cell Biol.* **142**, 373–388. 10.1007/s00418-014-1234-x (2014). 24981556 10.1007/s00418-014-1234-x

[40] Liu, C. F. & Lefebvre, V. The transcription factors SOX9 and SOX5/SOX6 cooperate genome-wide through super-enhancers to drive chondrogenesis. *Nucleic Acids Res.* **43**, 8183–8203. 10.1093/nar/gkv688 (2015). 26150426 10.1093/nar/gkv688 PMC4787819

[41] Akiyama, H. et al. Interactions between Sox9 and beta-catenin control chondrocyte differentiation. *Genes Dev.* **18**, 1072–1087. 10.1101/gad.1171104 (2004). 15132997 10.1101/gad.1171104 PMC406296

[42] Haseeb, A. et al. SOX9 keeps growth plates and articular cartilage healthy by inhibiting chondrocyte dedifferentiation/osteoblastic redifferentiation. *Proc. Natl. Acad. Sci. U. S. A.*10.1073/pnas.2019152118 (2021). 33597301 10.1073/pnas.2019152118 PMC7923381

[43] Zhang, Q. et al. SOX9 is a regulator of ADAMTSs-induced cartilage degeneration at the early stage of human osteoarthritis. *Osteoarthritis Cartilage* **23**, 2259–2268. 10.1016/j.joca.2015.06.014 (2015). 26162802 10.1016/j.joca.2015.06.014

[44] Song, H. & Park, K. H. Regulation and function of SOX9 during cartilage development and regeneration. *Semin. Cancer Biol.* **67**, 12–23. 10.1016/j.semcancer.2020.04.008 (2020). 32380234 10.1016/j.semcancer.2020.04.008

[45] Zhang, L. et al. LGALS3BP/Gal-3 promotes osteogenic differentiation of human periodontal ligament stem cells. *Arch. Oral Biol.* **128**, 105149. 10.1016/j.archoralbio.2021.105149 (2021). 34052527 10.1016/j.archoralbio.2021.105149

[46] He, X., Ohba, S., Hojo, H. & McMahon, A. P. AP-1 family members act with Sox9 to promote chondrocyte hypertrophy. *Development* **143**, 3012–3023. 10.1242/dev.134502 (2016). 27471255 10.1242/dev.134502 PMC5004882

[47] Topol, L., Chen, W., Song, H., Day, T. F. & Yang, Y. Sox9 inhibits Wnt signaling by promoting beta-catenin phosphorylation in the nucleus. *J. Biol. Chem.* **284**, 3323–3333. 10.1074/jbc.M808048200 (2009). 19047045 10.1074/jbc.M808048200 PMC2631972

[48] Li, V. S. et al. Wnt signaling through inhibition of beta-catenin degradation in an intact Axin1 complex. *Cell* **149**, 1245–1256. 10.1016/j.cell.2012.05.002 (2012). 22682247 10.1016/j.cell.2012.05.002

[49] Snelling, S. Chondrocyte isolation from human cartilage. (2019). 10.17504/protocols.io.8uphwvn

[50] Tchieu, J. et al. NFIA is a gliogenic switch enabling rapid derivation of functional human astrocytes from pluripotent stem cells. *Nat. Biotechnol.* **37**, 267–275. 10.1038/s41587-019-0035-0 (2019). 30804533 10.1038/s41587-019-0035-0 PMC6591152

[51] Wang, W. & Malcolm, B. A. Two-stage PCR protocol allowing introduction of multiple mutations, deletions and insertions using QuikChange Site-Directed Mutagenesis. *BioTechniques* **26**, 680–682. 10.2144/99264st03 (1999). 10343905 10.2144/99264st03

[52] Mayor, C. B. et al. Dubchak, I. VISTA: visualizing global DNA sequence alignments of arbitrary length. *Bioinformatics* **16**, 1046–1047. 10.1093/bioinformatics/16.11.1046 (2000). 11159318 10.1093/bioinformatics/16.11.1046

[53] Frazer, K. A., Pachter, L., Poliakov, A., Rubin, E. M. & Dubchak, I. VISTA: Computational tools for comparative genomics. *Nucleic Acids Res.* **32**, W273-279. 10.1093/nar/gkh458 (2004). 15215394 10.1093/nar/gkh458 PMC441596

[54] Rauluseviciute, I. R. P. et al. A. JASPAR : 20th anniversary of the open-access database of transcription factor binding profiles. *Nucleic Acids Res* 5, D174-D182 (2024). 10.1093/nar/gkad1059 10.1093/nar/gkad1059 PMC10767809 37962376

[55] Kaltner, H., Caballero, G. G. & Schmidt, S. Analysis of chicken LGALSL (galectin-related protein) gene’s proximal promoter and its control by Kruppel-like factors 3 and 7. *Gene* **933**, 148972. 10.1016/j.gene.2024.148972 (2025). 39343186 10.1016/j.gene.2024.148972

